# Supplementary material for: Mechanisms governing the pioneering and redistribution capabilities of the non-classical pioneer PU.1
Source: Nat Commun. 2020 Jan 21;11:402. doi: 10.1038/s41467-019-13960-2 (PMC6972792; doi:10.1038/s41467-019-13960-2)
Supplement: Supplementary file 7 — Source data [file 41467_2019_13960_MOESM7_ESM.zip › Source_Data/FigureS2/FigureS2B_MotifScanOutput/KmeansCluster7/homerResults/motif1.similar.html]

motif1

## Information for motif1

C
G
T
A
C
G
T
A
C
G
T
A
T
C
A
G
T
C
G
A
T
C
A
G
C
A
T
G
C
G
T
A
C
G
T
A
T
A
C
G
G
A
C
T
  
Reverse Opposite:  

C
T
G
A
A
T
G
C
G
C
A
T
G
C
A
T
G
T
A
C
A
G
T
C
A
G
C
T
A
G
T
C
G
C
A
T
G
C
A
T
G
C
A
T
  

|  |  |
| --- | --- |
| p-value: | 1e-1729 |
| log p-value: | -3.983e+03 |
| Information Content per bp: | 1.571 |
| Number of Target Sequences with motif | 2659.0 |
| Percentage of Target Sequences with motif | 82.83% |
| Number of Background Sequences with motif | 4729.7 |
| Percentage of Background Sequences with motif | 10.82% |
| Average Position of motif in Targets | 101.3 +/- 37.9bp |
| Average Position of motif in Background | 101.6 +/- 77.4bp |
| Strand Bias (log2 ratio + to - strand density) | -0.0 |
| Multiplicity (# of sites on avg that occur together) | 1.45 |
| Motif File: | file (matrix) reverse opposite |

### Similar de novo motifs found

|  |  |  |  |  |  |  |  |
| --- | --- | --- | --- | --- | --- | --- | --- |
| Rank | Match Score | Redundant Motif | P-value | log P-value | % of Targets | % of Background | Motif file |
| 1 | 0.982 | C T G A C G T A C G T A T C A G T C G A C T A G T A C G G T C A C G T A T A C G G A C T C T A G | 1e-1719 | -3958.445184 | 84.02% | 11.62% | motif file (matrix) |
| 2 | 0.968 | T C G A A T G C A C G T G A C T T A G C A G T C A G C T A G T C G C A T G C A T | 1e-1621 | -3732.694209 | 83.02% | 12.33% | motif file (matrix) |
| 3 | 0.965 | C T G A C T G A C T G A C G T A T C A G T C G A C T A G A C T G C G T A C G T A T A C G G A C T C T A G | 1e-1600 | -3685.589527 | 83.58% | 12.95% | motif file (matrix) |
| 4 | 0.923 | A G T C A G T C C G T A A T G C G A C T A C G T A G T C A G T C A G C T A G T C G A C T G A C T A G C T A G C T | 1e-1409 | -3244.641774 | 79.78% | 13.41% | motif file (matrix) |
| 5 | 0.957 | C G T A T C A G T C G A T C A G A C T G C G T A C G T A A T C G G A C T | 1e-1320 | -3040.931450 | 75.73% | 12.34% | motif file (matrix) |
| 6 | 0.944 | G A C T C T G A A T G C C G A T A G C T A G T C A G T C G A C T G A T C G C A T G C A T G C T A G A T C | 1e-1274 | -2935.663508 | 88.19% | 22.14% | motif file (matrix) |
| 7 | 0.950 | T C G A C G T A C G T A T A C G T C G A T C A G T C A G G C T A G C T A T A C G | 1e-1252 | -2883.154301 | 88.04% | 22.50% | motif file (matrix) |
| 8 | 0.955 | C G T A C G T A C G T A T C A G C T G A C T A G C T A G C G T A C G T A T A C G G A C T C G T A | 1e-1245 | -2867.093327 | 92.71% | 27.38% | motif file (matrix) |
| 9 | 0.915 | T C A G C T G A A C T G A C T G C G T A C G T A T A C G G A C T | 1e-1238 | -2852.104703 | 83.33% | 18.84% | motif file (matrix) |
| 10 | 0.945 | A G C T G A T C C G T A A T G C G C A T A C G T A G T C G A T C G A C T A G T C G C T A G T C A G C T A T G C A | 1e-1106 | -2546.760579 | 80.78% | 19.58% | motif file (matrix) |
| 11 | 0.888 | C T G A A C T G A C T G C G T A C G T A A T C G A C G T | 1e-1018 | -2344.205722 | 70.93% | 14.54% | motif file (matrix) |
| 12 | 0.907 | C T A G T C A G C T A G A T C G T C G A C G T A T A C G G A C T C T A G T G A C | 1e-981 | -2260.420703 | 75.89% | 18.61% | motif file (matrix) |
| 13 | 0.900 | A C T G T C G A T C A G A T C G G C T A C T G A T A C G C G A T C T A G | 1e-946 | -2178.710845 | 83.89% | 26.07% | motif file (matrix) |
| 14 | 0.887 | G A T C C T G A A G T C A C G T A G C T A G T C A G T C A G C T A G C T G A C T | 1e-938 | -2161.090791 | 88.26% | 30.67% | motif file (matrix) |
| 15 | 0.884 | A C T G C T G A A C T G A C T G C G T A C G T A A T C G | 1e-919 | -2117.794542 | 78.94% | 22.42% | motif file (matrix) |
| 16 | 0.845 | T C G A C G T A C T G A T C A G T C G A T C A G T C A G C T G A C T G A | 1e-911 | -2097.821904 | 63.93% | 12.36% | motif file (matrix) |
| 17 | 0.910 | T C A G C T G A C T A G A C T G C G T A C T G A T A C G G C A T C T G A T C A G T G C A | 1e-857 | -1975.541908 | 81.50% | 26.28% | motif file (matrix) |
| 18 | 0.826 | A T C G C G A T C G A T G A T C A G T C A G T C A G T C G C A T C A G T A G C T A G C T | 1e-845 | -1946.041201 | 51.93% | 7.46% | motif file (matrix) |
| 19 | 0.790 | A C G T A C G T A G T C A G T C A C T G A G T C C G T A A C G T | 1e-786 | -1811.781732 | 59.78% | 12.35% | motif file (matrix) |
| 20 | 0.812 | A T G C C G T A A T G C A C G T A C G T A G T C A G C T A G T C | 1e-768 | -1769.941077 | 77.76% | 25.51% | motif file (matrix) |
| 21 | 0.834 | G A C T T C A G A G T C C G A T A C G T A T G C A G T C A C G T T A G C A G C T G A T C | 1e-645 | -1487.454808 | 50.87% | 10.25% | motif file (matrix) |
| 22 | 0.762 | T G A C G A T C A C G T A G C T A G T C A G T C G A T C G A T C C A G T A C G T A C G T A G T C | 1e-480 | -1107.125357 | 38.16% | 7.11% | motif file (matrix) |
| 23 | 0.858 | C T G A A T G C G C A T A C G T G T A C A G T C G T A C G A T C G C A T G C A T G A C T | 1e-479 | -1103.065315 | 43.24% | 9.68% | motif file (matrix) |
| 24 | 0.852 | A T C G C T G A A T G C A C G T A C G T G A T C A G T C A G T C C A G T | 1e-274 | -632.625814 | 62.06% | 30.46% | motif file (matrix) |
| 25 | 0.785 | C T G A T A G C A C G T A C G T A G T C C G A T T A C G A G T C G C A T G C A T G C A T G C A T | 1e-191 | -441.278788 | 15.26% | 2.51% | motif file (matrix) |
| 26 | 0.753 | C G T A A T C G A C T G T C G A C T G A A T C G A G C T T G C A G T A C A G C T C G T A | 1e-175 | -405.108501 | 14.45% | 2.46% | motif file (matrix) |
| 27 | 0.777 | C A G T C T A G A C T G C G A T A G C T T A G C G A T C A C G T | 1e-156 | -359.287784 | 34.95% | 15.03% | motif file (matrix) |
| 28 | 0.755 | C T G A A C T G A C T G A C G T A C G T A G T C G A T C A C G T A T C G G A C T C G A T C A G T A G C T A C G T | 1e-100 | -231.610204 | 7.10% | 0.95% | motif file (matrix) |
| 29 | 0.752 | C G A T A C T G C A T G A C G T G C A T A G T C A G T C G A C T A G T C | 1e-63 | -146.032375 | 10.28% | 3.31% | motif file (matrix) |
